# Multidisciplinary Approach for the Management of a Case With Craniofacial Penetrating Injury Compressing the Internal Carotid Artery

**DOI:** 10.7759/cureus.37340

**Published:** 2023-04-09

**Authors:** Ayami Hamamoto, Tetsuhiko Michida, Tomoya Kawabata, Ryu Fukumitsu, Shogo Shinohara

**Affiliations:** 1 Department of Otolaryngology - Head and Neck Surgery, Kobe City Medical Center General Hospital, Kobe, JPN; 2 Department of Plastic Surgery, Kobe City Medical Center General Hospital, Kobe, JPN; 3 Department of Neurological Surgery, Kobe City Medical Center General Hospital, Kobe, JPN

**Keywords:** internal carotid artery, craniofacial penetrating injury, foreign body, multidisciplinary approach, ica injury, head injury, penetrating injury

## Abstract

A craniofacial penetrating injury can be severe when a foreign object reaches the skull base, causing an intracranial hemorrhage or a pseudoaneurysm. We report a case of sharp craniofacial injury in which a thin wooden rod moved from the orbit to the internal carotid artery. With a multidisciplinary team consisting of neurosurgeons, plastic surgeons, and otolaryngologists, the foreign body was safely removed, and the patient healed without complications or sequelae. Careful risk management is necessary when treating a case of craniofacial penetrating injury because the depth of the foreign body cannot be determined from the external appearance, making it challenging to decide on the severity of the damage from the injury.

## Introduction

Penetrating head injuries caused by foreign bodies are relatively rare, accounting for only 0.4% of all head injuries [1]. However, they have been reported to be especially dangerous when located at the middle skull base and can occasionally be lethal because of damage to the internal carotid artery (ICA) or the cavernous sinus [2]. Moreover, patients may be initially asymptomatic but present with severe central nervous deficiency, such as seizures or loss of consciousness for several days, months, or even years after the injury [3-5].

Therefore, craniofacial penetrating injuries require case-specific treatment organized by a multidisciplinary team depending on the route, material, and depth of damage caused by the injury, especially when it reaches the skull base [6]. Here, we report a case of a craniofacial penetrating injury caused by a thin wooden rod inserted from the medial wall of the orbit, passing through the nasal cavity, and compressing the ICA, which was successfully treated by a multidisciplinary team consisting of an otolaryngologist, plastic surgeon, and neurosurgeon.

## Case presentation

A 67-year-old woman fell on the grassy ground after a bicycle saddle and her legs were entangled while riding a bicycle. She did not notice any facial injury at that time. The next day, her left eyelid became swollen and she could not open her eyes. She visited a nearby ophthalmologist who confirmed that the foreign body was stuck in the side of her eye socket. However, the depth was unknown, and they referred her to our hospital because the transfer to a general hospital with adequate imaging capabilities was preferred. The patient had no relevant medical history.

**Figure 1:**
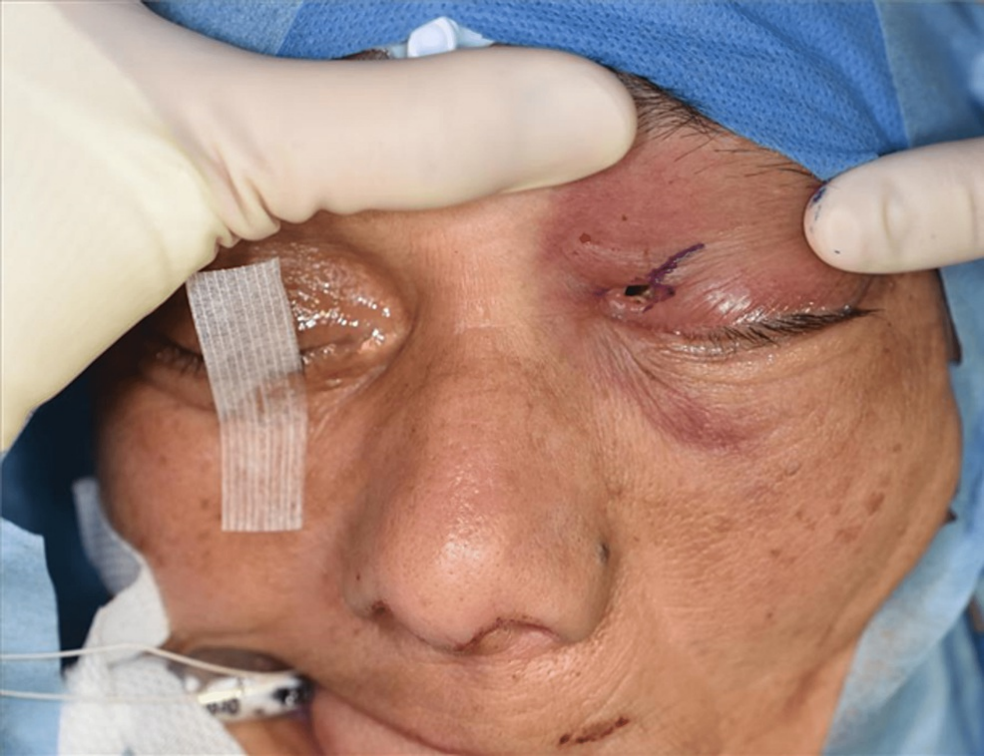
The left eyelid was erythematous and swollen, and a foreign body pierced precisely above the left medial canthus

**Figure 2:**
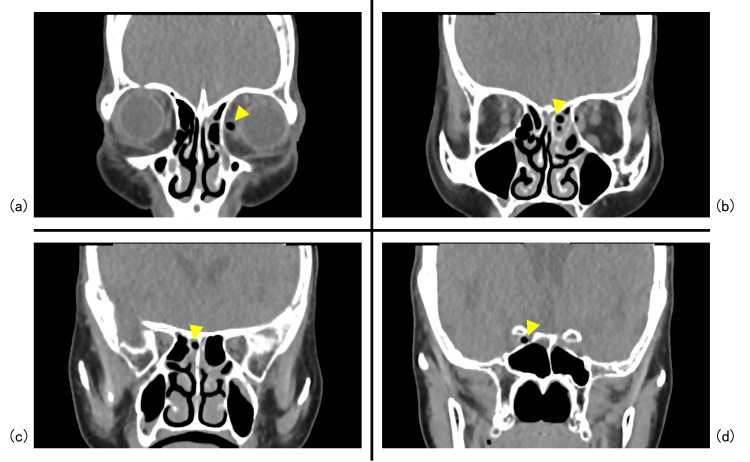
Preoperative coronal CT findings First, in the coronal section, the foreign body (yellow arrowhead) entered from the left medial orbit (a), passed through the left posterior ethmoid sinus (b) and right sphenoid sinus (c), and reached the vicinity of the right internal carotid artery (d).

**Figure 3:**
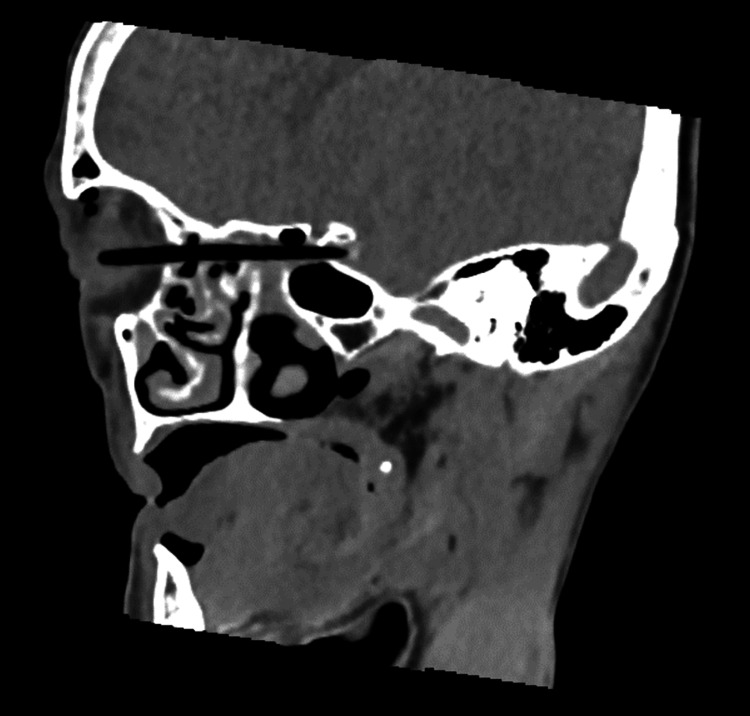
Preoperative sagittal CT findings The sagittal section clearly shows a continuous penetration pattern from the puncture point, rather than air.

**Figure 4:**
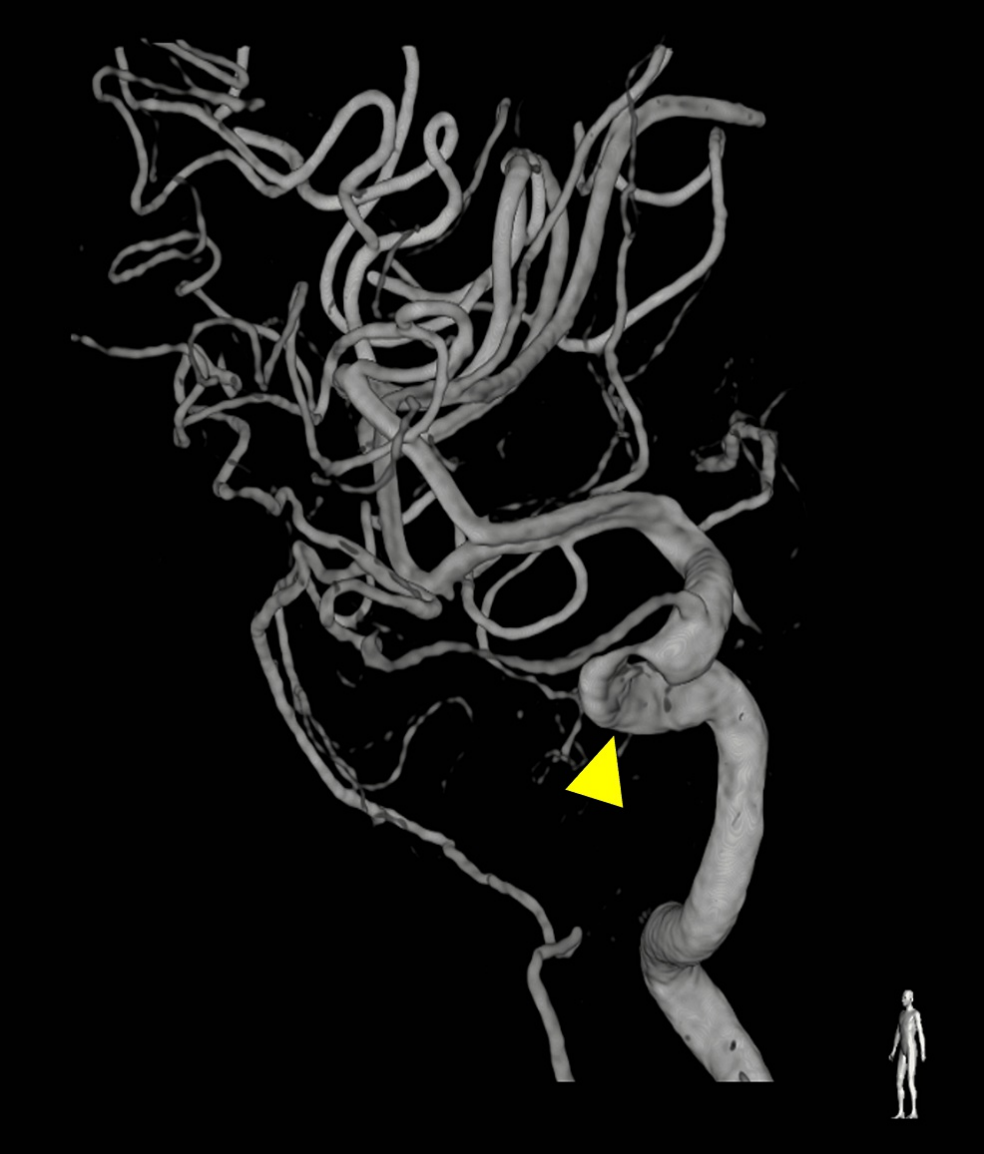
Preoperative digital subtraction angiography (DSA) The foreign body had compressed or penetrated the right internal carotid artery (yellow arrow).

When she came to our hospital, her Glasgow Coma Scale score was E4V5M6, she had no tetraplegia, and her vital signs were stable. The left eyelid was erythematous and swollen, and the puncture site of the foreign body was identified precisely above the medial canthus (Figure 1). Physical examination revealed no obvious spinal fluid leakage. Palpation revealed a puncture site on the body surface, but no foreign body was touching under the eyelids. The patient could not open her eyes independently, although her vision and visual acuity were normal. Blood tests indicated no elevated inflammatory response or anemia, and the patient did not have any other central nervous system deficiency. Computed tomography (CT) of the head revealed that the foreign body entered from the left medial orbit and passed through the left posterior ethmoid sinus and right sphenoid sinus. It penetrated the middle skull base (Figures 2, 3). Cerebral angiography was performed to determine the depth of the foreign body tip and possible storage of the ICA. The three-dimensional construction of contrast-enhanced images revealed that the right ICA was compressed or penetrated. Digital subtraction angiography (DSA)-CT suggested similar findings (Figure 4). However, it was unclear whether the foreign body penetrated the right ICA. Parent artery occlusion was necessary if the ICA was damaged during removal. Therefore, a balloon occlusion test was performed simultaneously to confirm that the patient was tolerant to occlusion of the right ICA. The emergency physician administered tetanus toxoid and tetanus immunoglobulin, a multidisciplinary team comprising otolaryngologists, plastic surgeons, and neurosurgeons was formed, and a therapeutic strategy was constructed to gather each member's roles.

**Figure 5:**
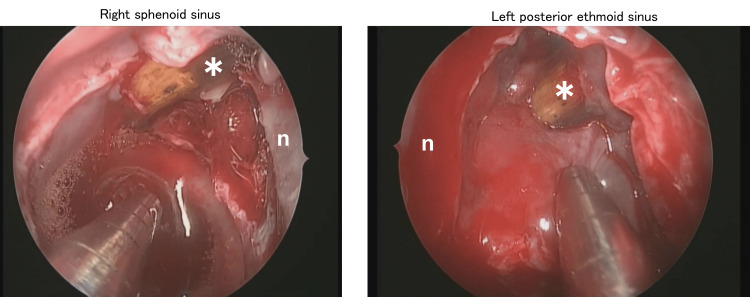
Intraoperative endoscopic findings A foreign body (*) was observed in the right sphenoid sinus and left posterior ethmoid sinus. n: nasal septum.

**Figure 6:**
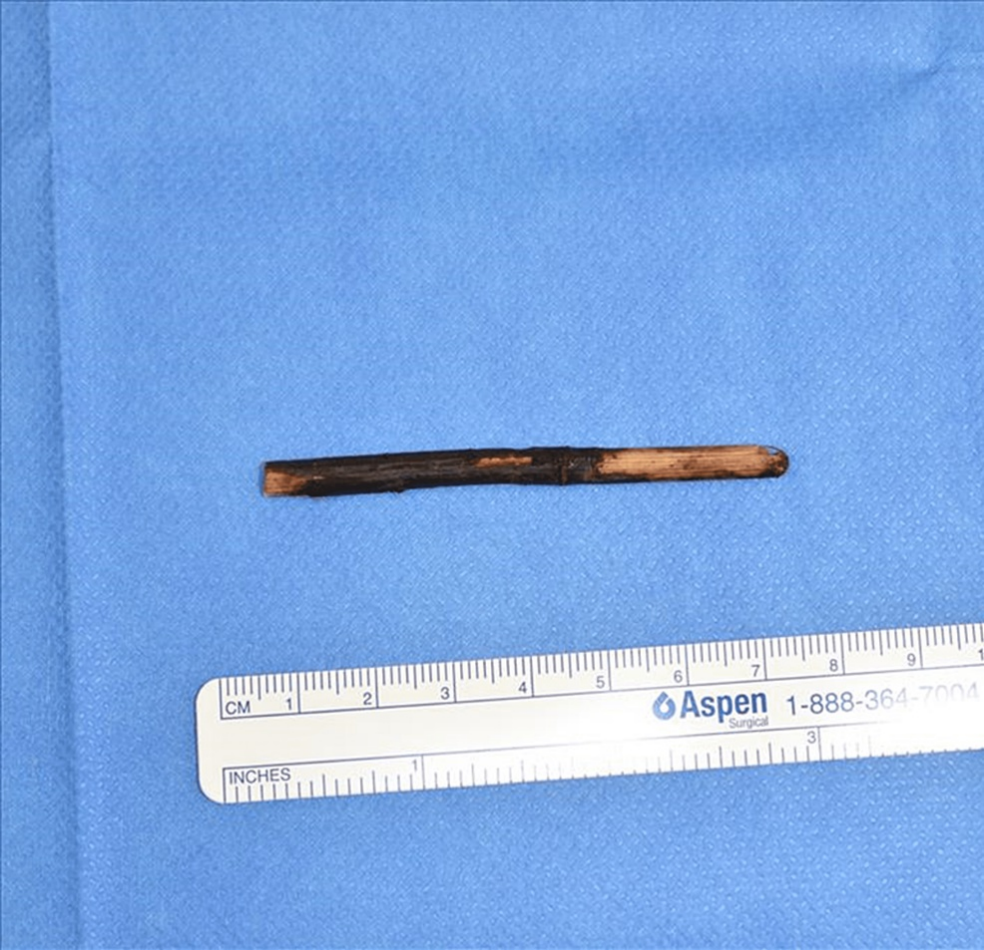
Foreign body A thin wooden rod approximately 7 cm in length.

The patient was moved to the hybrid operating room and the operation was initiated under general anesthesia. First, the otolaryngologists visualized the route of the foreign body in the nasal cavity using a navigation system by endoscopic sinus surgery, which came into the sinuses from the left ethmoid sinus to the right sphenoid sinus and penetrated the skull base posterior wall of the sphenoid sinus (Figure 5). They would play the role of temporary compression hemostasis and reconstruct the skull base in cases of massive bleeding or cerebrospinal fluid (CSF) leakage into the nasal cavity. Neurosurgeons placed a sheath inserted through the femoral artery on standby to allow immediate catheter stenting or embolization. The plastic surgeons made an incision, peeled it away from the face to increase the mobility of the wooden foreign body, and removed it. They prepared for the hematoma of the orbit and secured visual function. The removed foreign body was approximately 7 cm long (Figure 6). After removal, no massive hemorrhage or apparent CSF leakage was observed. DSA was performed again in the operating room to confirm that the ICA had been decompressed and that no apparent foreign body remained using an endonasal endoscope. The extraction hole (the entry point of the foreign body) was then washed with saline and filled with oxidized cellulose. Simultaneous cerebral angiography intraoperatively confirmed the absence of vascular injury.

After the surgery, the patient was kept at rest in a gatch bed up to 20 degrees until follow-up CT images were taken. Ceftriaxone (4 g/day) was administered to prevent postoperative meningitis, and the patient was discharged on the eighth postoperative day after no complications were confirmed, including postoperative infection. There were no findings suggestive of CSF leakage.

At the three-month follow-up examination, no evident CSF rhinorrhea was observed, and contrast-enhanced CT imaging revealed no aneurysm or other complications. The wound healed well with no visual field or vision deficiencies, and no eyeball protrusion. The patient was scheduled for a follow-up for aneurysm formation, meningitis, or other delayed complications.

## Discussion

Various types of foreign bodies are involved in penetrating injuries. Many studies have reported on penetrating injuries caused by foreign bodies made of wooden materials in Japan due to the forest environment and chopstick-using culture. Unlike metals or plastics, wooden materials are organic and porous and cause cellulitis or orbital cutaneous fistulas. Delayed diagnosis and treatment can lead to intracranial infections such as meningitis and brain abscesses [4,7]. When an intracranial penetrating injury is encountered, vital signs should be stabilized, and the entry and exit points of the puncture route should be identified to the extent possible. CSF leaks, intracranial hemorrhage, and loss of brain parenchyma are often not apparent in the external appearance and require imaging studies. Head CT imaging is the first choice because whether a metal fragment has strayed is unclear [8]. CT findings of wooden materials in the body vary over time and with the composition of the cellulose matrix [9]. In the acute phase, wooden materials demonstrate a low-density area on CT images resembling an air space in soft tissue. However, in the chronic stage, as the moisture content in the wooden materials increases, the concentration also increases [10]. Here, more than 24 hours had passed since the injury, and the foreign body was still observed as a low-density area.

Cases of traumatic ICA injury have often been reported in the literature, and the survival outcome of traumatic ICA occlusion is relatively poor, with a mortality rate of approximately 30-40%. By contrast, another 52% of the patients had severe neurological deficits [2,11,12]. Kazim et al. have recommended cerebral angiography in patients with penetrating head injuries with an increased risk of vascular injuries. They also emphasized the importance of angiography because unexpected subarachnoid hemorrhage or delayed hematoma may occur [8]. Katayama et al. reported a case of ICA injury caused by a penetrating head injury. They performed ICA trapping while removing the foreign bodies based on hemodynamics, as revealed by preoperative angiography. The patient demonstrated anopsia upon discharge, which was only due to the neurological deficit in this case [2]. Here, we performed a balloon occlusion test and confirmed that the patient was tolerant of occlusion of the unilateral ICA. In the case of an aneurysm or massive bleeding, the ICA would have instantly been stented or occluded by interventional radiology.

Two methods can be used to approach the skull base to remove foreign bodies causing penetrating head injuries: craniotomy and endoscopy. If the foreign body is completely intracranial, open surgery via craniotomy can be performed. Because of its better illumination and magnified visualization, the endoscopic approach has proven to be accurate for removing paranasal foreign bodies. Moreover, the endoscopic approach makes it possible to locate the entry route and confirm the presence of CSF leakage after the removal of a foreign body [13,14]. In cases of CSF leakage, a nasal septal mucoperiosteal flap could be efficiently utilized for repairing the skull base with a less invasive procedure [15].

The incidence of meningitis or brain abscess after removal has been reported in 1-5% of cases, and the probability increases when there is a CSF leak [8]. No apparent CSF leak was observed in this case; however, redness and swelling were observed at the entry site when the patient visited the hospital. Appropriate antibacterial medication and intraoperative lavage using saline may have helped prevent intracranial infection in the present case.

## Conclusions

Surgical treatment on a penetrating craniofacial injury from the medial wall of the orbit through the paranasal sinus to the ICA was successfully performed. It is often difficult to determine the severity of a penetrating wound because the depth of the wound is unclear on the outside, but as in this case, the CT image can be used to consider the relationship of the wound to the surrounding structures. In this case, the examination and surgical strategy planned by a multidisciplinary team contributed to the patient’s recovery without any complications or sequelae.
